# Experimental Study on the Characterization of Aging Resistance Properties of Optical Cables in the Hydrogen-Containing Downhole Environment

**DOI:** 10.3390/s24051655

**Published:** 2024-03-03

**Authors:** Feng Xu, Litong Li, Peng Jing, Zhiwei Yu, Jialiang Zhang, Shuhui Liu

**Affiliations:** 1 School of Information Engineering, Wuhan University of Technology, Wuhan 430073, China; xufengxf@whut.edu.cn (F.X.); zjliang@whut.edu.cn (J.Z.); 2 State Key Laboratory of Optical Fiber and Cable Manufacture Technology, Wuhan 430073, China; lilitong@yofc.com (L.L.); yuzhiwei@yofc.com (Z.Y.); 3 Yangtze (Wuhan) Optical System Limited Company, Wuhan 430073, China; 4 Hubei Key Laboratory of Optical Information and Pattern Recognition, Wuhan Institute of Technology, Wuhan 430205, China; 2210070303@stu.wit.edu.cn

**Keywords:** downhole optical cables, hydrogen penetration, hydrogen loss, aging of optical fibers

## Abstract

The utilization of downhole optical cables has significantly enhanced the efficiency and reliability of oilfield production operations; however, the challenging high-temperature and high-pressure conditions prevalent in oil-gas fields markedly reduce the service lifespan of these optical cables. This limitation severely impedes their application and further development in subterranean environments. In this study, a qualitative analysis was conducted on the structural materials utilized in two types of optical cables to identify these materials and assess the high-temperature tolerance and aging resistance properties of the optical fibers incorporated within. It was discovered that hydrogen infiltration into the subterranean optical cables predominantly accounts for their operational failure. To address this issue, an optical loss testing platform was established, facilitating the execution of a high-temperature and high-pressure hydrogen permeation aging experiment on the optical fibers, allowing for the evaluation of the hydrogen resistance capabilities of the two types of optical fibers. The findings from this study provide a theoretical foundation and methodological guidance for the optimization of optical fibers, aiming to enhance their durability and functional performance in adverse environmental conditions encountered in oil-gas field applications.

## 1. Introduction

In confronting the challenge of low oil prices and aiming to reduce costs while improving quality and efficiency, the global oil industry is advancing toward digitalization and intelligence. This shift necessitates the economic and efficient acquisition of downhole information from oil-gas wells. The advancements in optical fiber communication and sensing technologies have significantly enhanced the reliability and cost-effectiveness of downhole measurement and control systems. Optical fiber, with its attributes of immunity to electronic interference, corrosion resistance, ease of installation, and compact size, is ideally suited for the permanent monitoring of well conditions. Consequently, downhole multi-parameter monitoring using optical fiber has become the preferred method for information acquisition and well condition monitoring in the realm of intelligent well technology.

The high-temperature and high-pressure conditions prevalent in downhole environments, especially those containing hydrogen, present a formidable challenge to the long-term deployment of optical fibers. Due to the inherent low strength of the fiber, it is combined with various reinforcement structures such as organic gels, polymer materials, and metal reinforcements to construct optical cables suitable for use. Previous studies have demonstrated that temperature significantly influences the stability of fiber optic signal transmission [1,2,3,4]. In the 1980s, it was observed that optical fibers would experience signal attenuation when operated in environments containing hydrogen. Ning Ding et al. [5] conducted a theoretical analysis of the diffusion mechanism of hydrogen, confirming a hydrogen-induced loss and providing a foundation for the long-term reliable application of optical cables. Wang Derong et al. [6] examined the relationship between the vibration absorption loss of hydrogen molecules, time, temperature, and hydrogen partial pressure, as well as the effect of fiber optic ointment on hydrogen loss in optical cables. Xiao Xiaoming et al. [7] introduced methods to prevent hydrogen loss in optical cables and to recover from such losses. Wang Lintang et al. [8] reviewed the mechanisms and sources of hydrogen loss in optical fibers, offering crucial guidelines for the detection and assessment of hydrogen loss, thus laying the groundwork for evaluating hydrogen-induced attenuation in optical fibers. More recently, Stolov et al. investigated the impact of hydrogen-rich environments on fibers with cores made of pure silica and fluorine-doped and germanium-doped materials [9]. The coating of the fiber, which significantly influences its strength, has been the subject of extensive research and development, employing various materials such as aluminum, gold, and polyimide (PI) to enhance its resilience in the demanding conditions encountered in well environments [10]. Furthermore, advancements have been made in polyimide curing techniques aimed at enhancing the performance of carbon/polyimide-coated optical fibers in hydrogen-rich atmospheres [11]. However, much of the research conducted thus far has not extensively covered the long-term reliability and adaptability of optical fibers within the confined and extreme conditions of downhole environments, particularly lacking a comprehensive understanding of the anti-aging properties of optical cables subjected to high-temperature and high-pressure hydrogen permeation [12,13,14,15,16]. The primary challenge affecting the lifespan of optical fibers, namely, the signal attenuation in fiber optic sensing elements, remains insufficiently addressed. This issue is predominantly due to the increase in optical loss caused by the high temperature and hydrogen degradation of the fiber [17,18,19,20]. The infiltration of hydrogen into the core of both single-mode and multi-mode fibers can lead to significant complications, most notably extensive attenuation across a broad wavelength spectrum, which compromises the fiber’s efficiency in data transmission or sensing applications.

In this paper, considering the downhole environmental conditions such as high temperature, high pressure, and hydrogen exposure, an environmental durability test involving high-temperature and high-pressure oil–gas–water mixtures is conducted on optical cables. Furthermore, the optical fibers undergo a high-temperature and high-pressure hydrogen permeation aging process, during which their attenuation is assessed. By analyzing the loss of optical fiber power under varying conditions of temperature, pressure, and hydrogen partial pressure, the aging behavior of the optical fibers can be elucidated. The insights gained from this investigation can serve as valuable guidance for the design, manufacturing, and operational deployment of underground optical cables, ensuring their reliability and performance in harsh downhole environments.

## 2. Test Sample Selection and Performance Analysis

**Figure 1:**
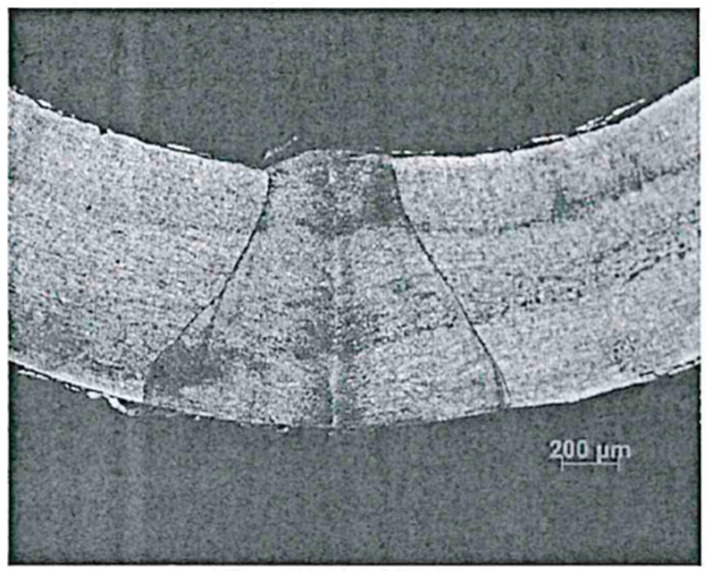
Microscopic Photographs of Optical Cable Welds.

**Figure 2:**
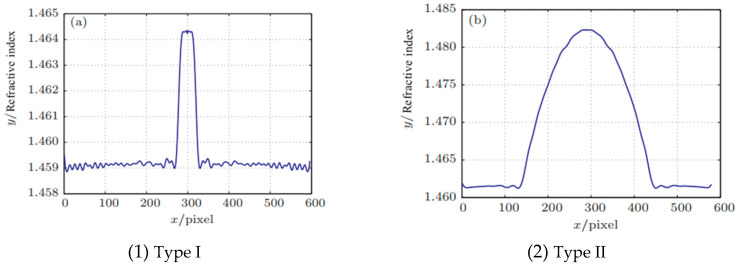
The Refractive Index Distribution of Different Optical Fibers. (**a**) The refractive index distribution along the diameter direction of a single-mode fiber along the cleavage plane; (**b**) The refractive index distribution along the diameter direction of a multi-mode fiber along the cleavage plane.

The standard optical cable and optical fiber for oil wells are selected, as shown in Table 1 and Figure 1. The refractive index distribution of different optical fibers is shown in Figure 2.

## 3. Test Process

The optical power loss (∆P) before and after fiber aging is utilized to quantify the degree of fiber aging. In this testing process, a stable light source is employed to output the optical signal of a specific wavelength, mode, and power. The optical power meter is then used to measure the received power. The output power of the stable light source is specified as 5 ± 0.2 dBm. For Type I fiber, which is a multi-mode fiber, the selected test wavelengths are 850 nm and 1300 nm, and it is categorized as “normal temperature fiber”. For Type II fiber, which is a single-mode fiber, the selected test wavelengths are 1310 nm and 1550 nm, and it is classified as “high-temperature fiber”. It is noted that the welding loss is relatively small, approximately around 0.05 dB.

The fiber optic hydrogen penetration test scheme and process are as follows:

The hydrogen permeation testing apparatus used in this study is a custom-designed hydrogen permeation reactor specifically for this test. By adjusting the temperature and hydrogen pressure of the reactor and controlling the hydrogen permeation time of the optical fiber, the purpose of accelerating the hydrogen permeation of the fiber is achieved.

**Figure 3:**
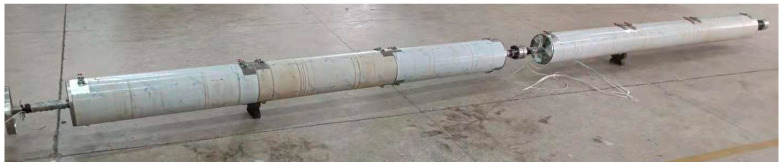
Hydrogen permeation test autoclave.

The hydrogen permeation apparatus is a 5-meter-long stainless steel closed-tube hydrogen permeation testing high-pressure autoclave reactor. The schematic diagram of the testing apparatus is shown in Figure 3. In addition to the hydrogen permeation chamber, the comprehensive hydrogen permeation apparatus also includes auxiliary equipment such as a nitrogen cylinder, hydrogen cylinder, pressure pump, booster pump, bursting valve, and temperature control box.

The inner diameter of the high-pressure autoclave is 20 mm, with a total length of 5 m. It consists of two 2.5-m-long stainless steel pipes connected by flanges and sealed with graphite rings. There are holes at both ends of the high-pressure autoclave for vacuum pumping and pressurization, with the other end sealed, and the sample placed in the middle section.

The fiber optic hydrogen permeation test involves a series of operations: collecting the optical fiber, loading the sample, placing it into the high-pressure autoclave, undergoing the aging process, and finally sampling. The initial steps involve acquiring samples that meet the experimental objectives, including obtaining specified lengths of optical cables or pure optical fibers. Due to the tendency of pure optical fibers to curl, careful unwinding is required after a transfer, especially challenging to obtain a 5-m-long segment of pure optical fiber. Due to the fragility of pure optical fibers and their susceptibility to damage, the fibers must be packaged in stainless steel tubes before the hydrogen permeation process to ensure the integrity of the fibers. Once encased in stainless steel, the samples are sealed with aluminum foil and glass fiber cloth for protection and then placed in the central portion of the high-pressure autoclave for experimentation. Optical cable samples protected by an external stainless steel layer are typically stored in a coiled state. To facilitate the insertion of these samples into the reactor, they must be extended as much as possible. Additionally, these samples are wrapped in a layer of polypropylene (PP) plastic packaging, which must be removed before the experiment begins. Neglecting this step could result in the plastic melting at temperatures around 200 °C, potentially affecting the experimental process.

During the pressurization and heating process, it is essential to raise the temperature to the target level before pressurizing. Pressurizing before heating will cause the pressure to increase beyond the target temperature. Conversely, during the depressurization and cooling process, it is essential to first lower the temperature and then reduce the pressure. During the sampling process, careful handling of the optical fiber is crucial as they are fragile and easily damaged. Subsequently, the pure optical fiber should be coiled and stored, and loss testing should be conducted. Extracting optical fiber from optical cables requires caution as the internal lubricant on the fibers has strong adhesive properties, and the length of the cable makes it impossible to extract the fiber completely. Therefore, it is necessary to cut the external stainless steel layer of the cable in sections rather than cutting it completely to ensure the integrity of the optical fiber. Following this procedure enables the complete extraction of the optical fiber sample.

**Figure 4:**
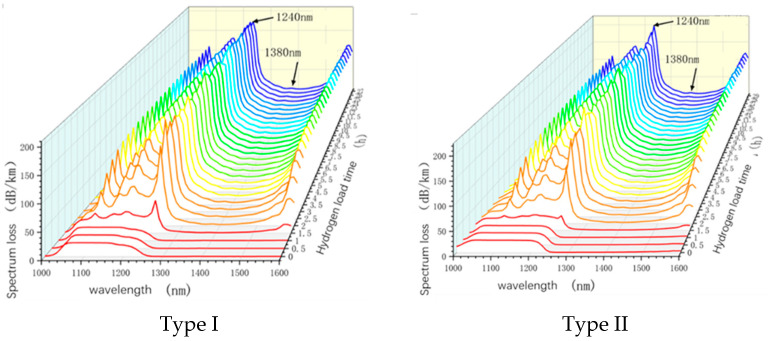
Spectrum loss test results of different optical fibers in a pure hydrogen environment.

To investigate the impact of a hydrogen environment on fiber optic losses, two types of optical fibers were selected for real-time monitoring of hydrogen-induced losses. The real-time loss values were specifically monitored at wavelengths of 1240 nm and 1380 nm. The wavelength of 1240 nm serves as a representative absorption wavelength for hydrogen molecules, where a higher value indicates a higher concentration of hydrogen molecules dissolved through diffusion in the fiber core. On the other hand, the wavelength of 1380 nm represents the characteristic absorption wavelength of -OH, indicating a higher value signifies a greater presence of -OH formed by the combination of non-bridging oxygen with hydrogen in the fiber core. The loss results are illustrated in Figure 4. Analysis of the spectral loss detection results of the two types of optical fibers reveals that with the passage of time, the peak values of the characteristic wavelengths at 1240 nm and 1380 nm gradually increase. This observation suggests that as the hydrogen loading time extends, hydrogen progressively diffuses into the fiber core.

On this basis, fiber aging and hydrogen loss tests under different conditions were carried out; the optical fiber aging conditions can be seen in Table 2.

## 4. Fiber Aging and Hydrogen Loss Test

In this experiment, the aging process of optical fiber was accelerated through high-temperature hydrogen pressure to investigate the signal loss during aging. The optical power loss of the fiber before and after aging was used as the evaluation index.

**Figure 5:**
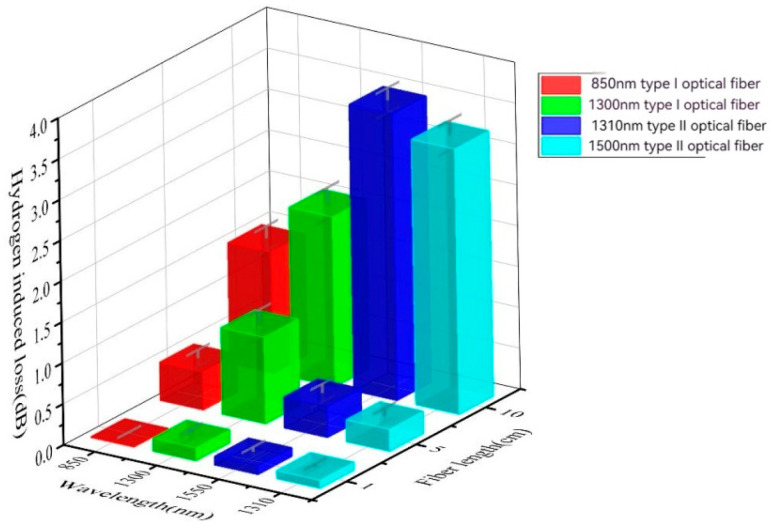
Hydrogen loss under pure H_2_, 2 MPa, 200 °C, and 96 h aging conditions.

Table 3 presents the variation in fiber loss with respect to the core length under aging conditions of pure H_2_, 2 MPa, 200 °C, and 96 h. Type I fiber refers to a multi-mode fiber with test wavelengths of 850 nm and 1300 nm, while Type II fiber refers to a single-mode fiber with test wavelengths of 1310 nm and 1550 nm. As shown in Figure 5, the loss of both types of fiber increases with the increase in core length, indicating that longer fiber aging leads to greater loss under the same conditions. In the pure H2 environment, Type I fiber exhibits a lower hydrogen loss compared to Type II fiber. Additionally, the test wavelength of the optical signal also influences the loss. For the same fiber type, higher wavelengths such as 1300 nm and 1550 nm result in greater sensitivity and higher loss. To maintain consistent conditions, a fiber length of 5 m was used in subsequent experiments.

**Figure 6:**
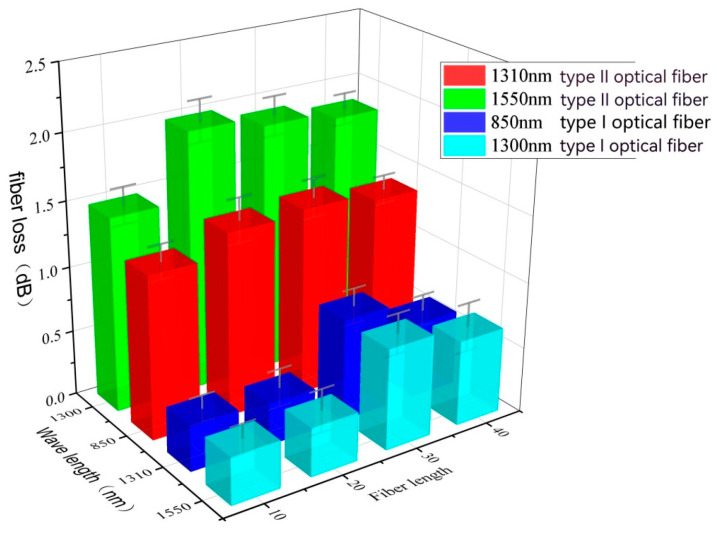
Different total pressure (H_2_ partial pressure 2 MPa), 200 °C, and hydrogen loss after 96 h.

Table 4 and Figure 6 illustrate the hydrogen loss after aging under different total pressures (H_2_ partial pressure 2 MPa) at 200 °C for 96 h. As the pressure increases, the loss of both types of fibers increases, reaching a peak at 30 MPa, after which the loss starts to decrease, but the overall change is not significant. It can be inferred that the hydrogen loss in the fibers is most severe at 30 MPa. With an increase in total pressure, the permeation rate of hydrogen accelerates. However, since the partial pressure of hydrogen remains unchanged, the difference in loss between each sample is not significant. The smaller loss observed in Type I fiber indicates better resistance to hydrogen permeation. Furthermore, for the same type of fiber, the loss increases with the increase in the wavelength of the optical signal, which is consistent with the previous results.

**Figure 7:**
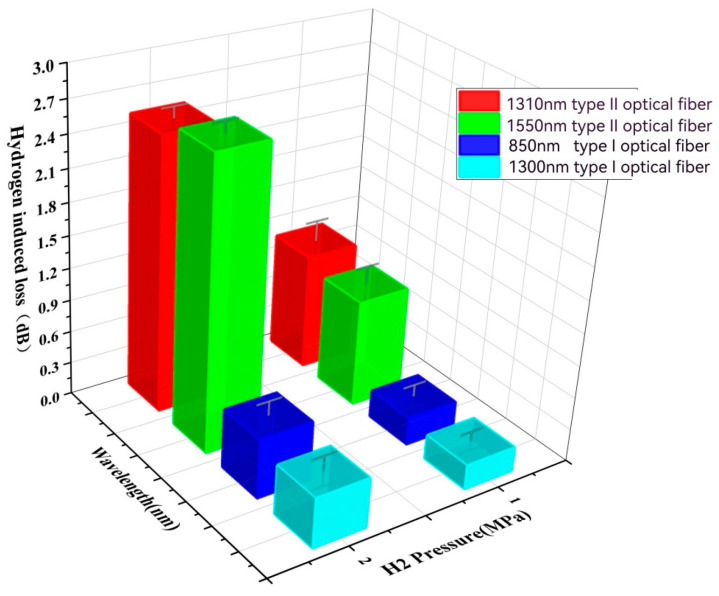
Hydrogen loss diagram after aging at 200 °C and 96 h for different H_2_ partial pressures.

Table 5 and Figure 7 present the hydrogen loss under aging conditions of total pressure at 10 MPa, H_2_ partial pressure at 1 MPa and 2 MPa, at 200 °C for 96 h. It is observed that with an increase in H2 partial pressure, the losses of both Type I and Type II fibers increase, although the loss value for Type I fiber remains relatively low.

**Figure 8:**
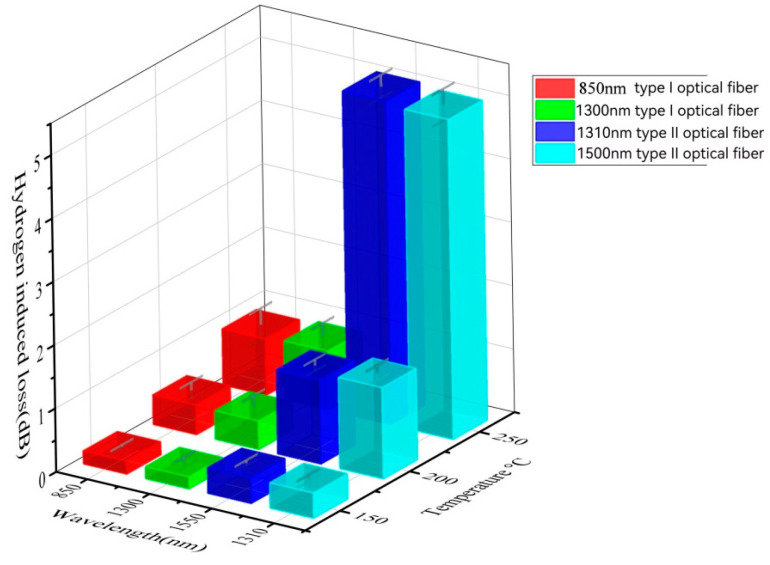
Hydrogen loss diagram after aging for 96 h under different temperatures at a total pressure of 10 MPa and a H_2_ pressure of 2 MPa.

Table 6 and Figure 8 depict the hydrogen loss under aging conditions of total pressure at 10 MPa, H2 partial pressure at 2 MPa, and different aging temperatures (150 °C, 200 °C, 250 °C) for a duration of 96 h. It is observed that with an increase in temperature, the loss of both types of fibers increases. Furthermore, at a high temperature of 250 °C, there is an accelerated aging trend, possibly due to the temperature-induced acceleration of surface hydrogen aging reactions, leading to an increased fiber loss. In comparison, Type I fiber maintains a loss value between 0.7 and 0.95, while the loss of Type II fiber exceeds 5. This indicates that Type I fiber exhibits better temperature resistance.

**Figure 9:**
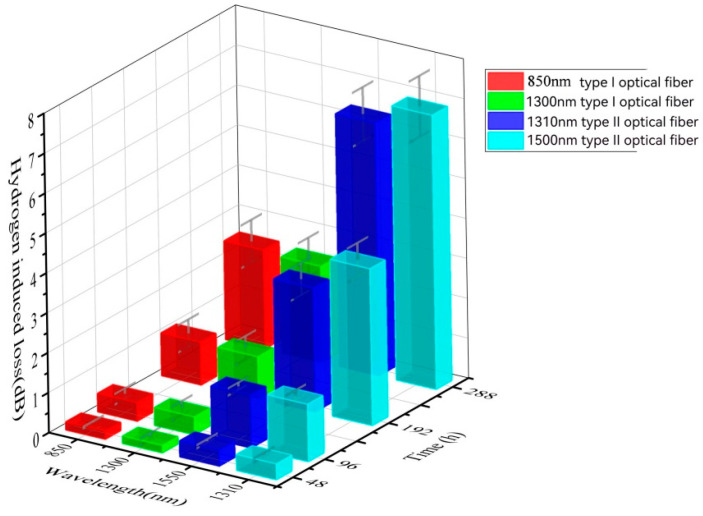
Hydrogen loss diagram after aging different times under 200 °C at a total pressure of 10 MPa and a H_2_ pressure of 2 MPa.

Table 7 and Figure 9 present the hydrogen loss under aging conditions of total pressure at 10 MPa, H2 partial pressure at 2 MPa, and aging temperature at 200 °C for various aging durations. It is evident that with prolonged aging time, the loss of both types of fibers increases. Moreover, aging becomes more pronounced as time progresses, likely due to the significant increase in surface hydrogen concentration with extended time, thus accelerating the occurrence of surface hydrogen loss. In comparison, Type I fiber still exhibits relatively good performance in terms of loss under these conditions.

## 5. Ointment-Coated Fiber Test

The fiber gel serves as a filler between the exposed portion of the fiber and the cable protective layer, providing protection to the bare fiber in two significant ways: firstly, effectively preventing moisture ingress, and secondly, acting as a cushioning layer against external mechanical impacts, maintaining the fiber in a free, low-stress state and buffering mechanical movements such as vibration and bending.

Both Type I and Type II fibers are immersed in the fiber gel environment and subjected to aging under high-temperature conditions inside a furnace. The experimental conditions remain consistent across all tests, with an atmospheric pressure of 250 °C and aging durations of 12 h, 24 h, 36 h, and 48 h. A control group without fiber gel is also established, with a duration of 24 h.

**Figure 10:**
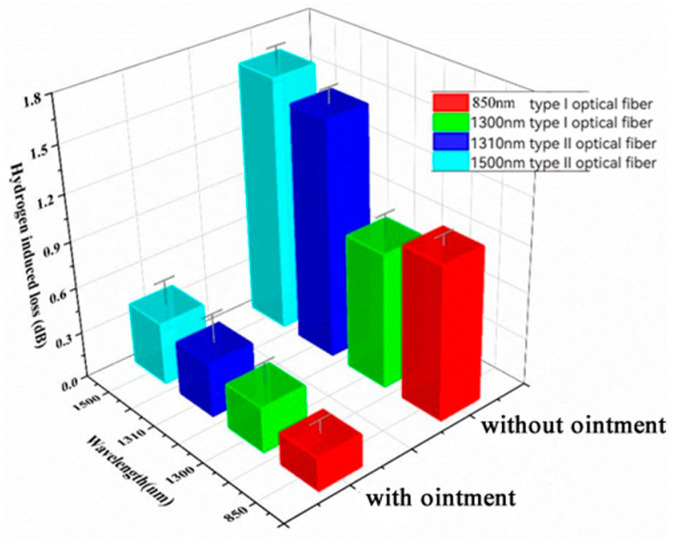
Table diagram of hydrogen loss after aging for 24 h with or without ointment package (atmospheric air at 250 °C).

**Figure 11:**
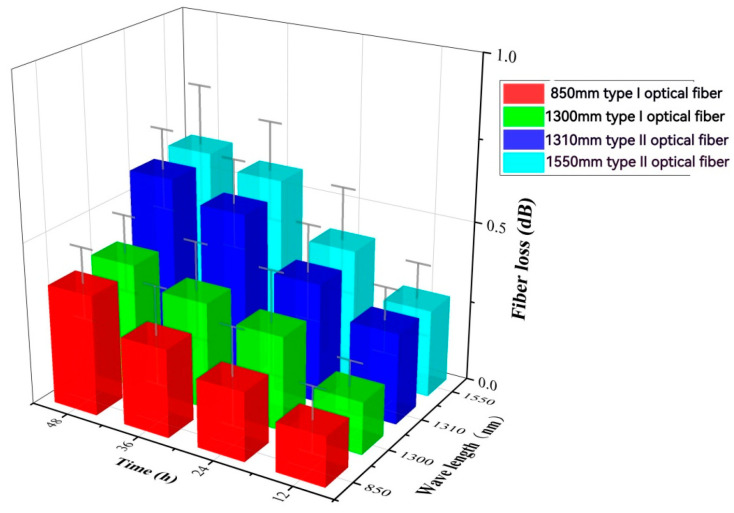
Hydrogen loss table diagram of aging times on optical fiber loss (ointment, atmospheric air at 250 °C).

The same type of optical gel was applied to two types of optical fibers and subjected to heating under different aging conditions in a furnace to investigate the patterns. Table 8 presents the hydrogen loss under varying aging times at atmospheric pressure and a temperature of 250 °C with the protection of the same optical gel. Figure 10 illustrates the hydrogen loss after 24 h of aging (at atmospheric pressure in air at 250 °C) with and without optical gel wrapping as control groups. A comparison of fiber loss with and without optical gel shows that the gel provides a certain level of protection, significantly reducing fiber loss. Figure 11 displays the hydrogen loss of fiber loss under different aging times (optical gel, atmospheric pressure in air at 250 °C), indicating a decrease in the protective effectiveness of the optical gel with increasing aging time, leading to continuous fiber loss. Additionally, from Table 8, it can be observed that the fiber loss after 48 h with optical gel is significantly lower than the fiber loss after 24 h without optical gel, demonstrating that the optical gel continues to protect the fibers even after 48 h of aging. Furthermore, it is noticeable that the fiber loss of Type I fibers consistently remains significantly lower than that of Type II fibers, indicating the superior high-temperature resistance of Type I fibers over Type II fibers.

## 6. Conclusions

This study conducted high-temperature and high-pressure hydrogen permeation aging tests on optical fibers, and the results showed that, under pure H_2_ aging conditions, the attenuation of both types of fibers increased with the increase in fiber length. In a pure H2 environment, Type I fibers exhibited lower hydrogen-induced losses compared to Type II fibers. Under different total voltage aging conditions, the losses of both types of fibers increased with increasing pressure, reaching a peak at 30 megapascals, and then began to decrease. The attenuation of both Type I and Type II fibers increased with increasing H_2_ partial pressure, indicating a significant influence of H_2_ concentration on fiber loss. An enhanced H_2_ partial pressure accelerated the permeation of hydrogen atoms into the fibers, resulting in increased fiber loss.

With increasing temperature and prolonged exposure time, the losses of both Type I and Type II fibers increased, but the hydrogen permeation loss of Type I fibers was superior to that of Type II fibers. Wrapping the same type of optical gel on the surfaces of both types of fibers and subjecting them to atmospheric pressure heating and aging experiments demonstrated that the optical gel provided a certain level of protection and significantly reduced fiber loss.

This paper lays the foundation for the optimal selection of fibers and related materials. By conducting aging tests on optical cables and fibers, the resistance performance of the two types of fibers to high-pressure and high-temperature hydrogen permeation was evaluated. These evaluations provide valuable insights into the design, processing, manufacturing, utilization, and maintenance of underground optical cables.

## Figures and Tables

**Table 1:** Specifications of Optical Cable Samples for Oil wells.

Optical Cable Cross-Sectional Diagram			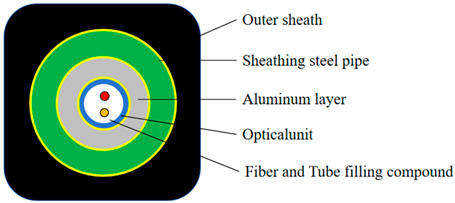		
Inspection Item			Unit	Standard Requirements	Results of Inspection
fiber length and attenuation	SMF (orange)	length	m	/	1002
		attenuation@1310 nm	dB/km	≤0.5	0.327
		attenuation@1550 nm	dB/km	≤0.4	0.187
	MMF (blue)	length	m	/	1002
		attenuation@1310 nm	dB/km	≤4.0	2.480
		attenuation@1550 nm	dB/km	≤2.0	0.667
fiber unit	outer diameters		mm	2.4 ± 0.05	2.40
	wall thickness		mm	0.2 ± 0.02	0.20
aluminum coating	outer diameters		mm	4.4 ± 0.05	4.38
	wall thickness		mm	1.0 ± 0.05	0.98
protective layer steel pipe	outer diameters		mm	6.35 ± 0.05	6.34
	wall thickness		mm	0.89 ± 0.05	0.89
	ovality		mm	±5% outer diameter	0.02
welding integrity of protective layer steel pipe	weld fiber photos		/	The microstructure of the weld should be full, symmetrical in shape, and without welding and forming defects.	See attached Figure 1
	beading		%	The outer diameter expansion value is not less than 30%, and there is no cracking in the weld.	Expanding to 38%, the weld has no cracking.
	flattening		mm	When H ≤ 4.21 mm, there is no cracking in the weld.	When H = 2.13 mm, there is no cracking in the weld.
	curling		%	The hemming width is not less than 15% of the outer diameter, there is no cracking in the weld.	Edge curling up to 40%, weld no cracking
	reverse flattening		/	After the sample is completely flattened, there are no defects such as cracks at the weld.	pass
	eddy current testing		/	Should meet the acceptance level EH4	pass
breaking force	/		kN	≥8	12.9
remaining length	/		‰	≥3.0	3.5
unit weight	/		kg/km	245 ± 3	243
boundary dimension	/		mm	8.8 × 8.8	8.6 × 8.7
optical fiber cable length	/		m	/	500

**Table 2:** Optical fiber aging conditions.

Total Pressure (MPa)	Partial Pressure of H_2_ (MPa)	Temperature (°C)	Time (h)
10	1	200	96
10	2		
20	2		
30	2		
40	2		
2	2		
10	2	150	96
10	2	250	96
10	2	200	48
10	2	200	192
10	2	200	288

**Table 3:** Hydrogen loss under pure H_2_, 2 MPa, 200 °C, and 96 h aging conditions.

Length of Optical Fiber (m)	Type I		Type II	
	850 nm	1300 nm	1310 nm	1550 nm
1	0.01	0.16	0.11	0.1
5	0.47	1.06	0.38	0.27
10	1.48	2.08	3.51	3.25

**Table 4:** Different total pressure (H_2_ partial pressure 2 MPa), 200 °C, and hydrogen loss after 96 h aging.

Pressure (MPa)	Type I		Type II	
	850 nm	1300 nm	1310 nm	1550 nm
10	0.35	0.36	1.24	1.48
20	0.32	0.37	1.4	1.88
30	0.78	0.77	1.43	1.9
40	0.58	0.65	1.36	1.84

**Table 5:** Hydrogen loss table after aging at 200 °C and 96 h for different H_2_ partial pressures.

Pressure (MPa)	Type I		Type II	
	850 nm	1300 nm	1310 nm	1550 nm
1	0.26	0.23	1.08	0.99
2	0.55	0.46	2.53	2.67

**Table 6:** Hydrogen loss table after aging 96 h under different temperatures at a total pressure of 10 MPa and a H_2_ pressure of 2 MPa.

Temperature (°C)	Type I		Type II	
	850 nm	1300 nm	1310 nm	1550 nm
**150**	0.21	0.19	0.41	0.4
**200**	0.47	0.45	1.33	1.44
**250**	0.89	0.93	5.11	5.05

**Table 7:** Hydrogen loss table after aging different times under 200 °C at a total pressure of 10 MPa and a H_2_ pressure of 2 MPa.

Time (h)	Type I Communication Optical Fiber		Type II Communication Optical Fiber	
	850 nm	1300 nm	1310 nm	1550 nm
**48**	0.24	0.27	0.53	0.51
**96**	0.47	0.45	1.33	1.44
**192**	1.08	1.05	3.11	3.93
**288**	2.48	2.41	6.34	6.87

**Table 8:** Hydrogen loss table of atmospheric air aged at 250 °C for different times.

Ointment	Time (h)	Type I		Type II	
		850 nm	1300 nm	1310 nm	1550 nm
-	0	0.14	0.12	0.21	0.24
Y	12	0.15	0.16	0.28	0.27
Y	24	0.21	0.29	0.37	0.40
N	24	0.99	0.87	1.53	1.64
Y	36	0.27	0.33	0.53	0.57
Y	48	0.37	0.39	0.59	0.61

## Data Availability

The data presented in this study are available on request from the corresponding author.
